# Water of Copaiba for Injections

**Published:** 1846-10

**Authors:** 


12. *Water of Copaiba for Injections.*—Dr. Cattell recommends the following formula for the preparation of this article. ℞. Ol. Copaibæ, two ounces; magnesiæ carb., six drachms. Rub together and add four gallons, or less, of water. Filter. Cubebs may be prepared in the same way. This preparation is employed by injection in those cases, where the article is indicated, such as gonorrhœa, lencorrhœa, &c. By using injections, the nauseating effect of the remedy is avoided.—*West. Lancet*, in *Southern Med. and Surg. Journal.*

